# How important is gametocyte clearance after malaria therapy?

**DOI:** 10.1186/s12916-016-0641-3

**Published:** 2016-06-18

**Authors:** Harin A. Karunajeewa, Ivo Mueller

**Affiliations:** Walter and Eliza Hall Institute of Medical Research, 1G Royal Parade, Parkville, Vic 3052 Australia; Western Centre for Health Research and Education, Western Health, Melbourne, Australia; Malaria: Parasites & Hosts Unit, Institut Pasteur, Paris, France

**Keywords:** Malaria, *P. falciparum*, Antimalarial, Gametocytes, Gametocyte clearance, Artemisinin combination therapy

## Abstract

There has been increasing interest in the role of malaria drugs in preventing malaria transmission from humans to mosquitoes, which would help augment malaria control and elimination strategies. Nevertheless, only one stage in the malaria parasite life cycle, the gametocyte, is infectious to mosquitoes. The Worldwide Antimalarial Resistance Network (WWARN) have analyzed data from 48,840 patients from 141 clinical trials in order to define the nature and determinants of gametocyte clearance following artemisinin combination treatment (ACT) for symptomatic malaria infections. However, the presence of gametocytes does not always predict their infectivity, meaning that the microscopy-based methods used by the WWARN investigators represent an imperfect surrogate marker of transmissibility. Their findings, that some ACTs clear gametocytes faster than others, should be interpreted in light of these limitations and important gaps in our understanding of the biology and epidemiology of malaria transmission.

Please see related article: https://bmcmedicine.biomedcentral.com/articles/10.1186/s12916-016-0621-7

## Background

Global improvements in malaria control over the last 15 years have been greater than at any other time in history, with annual deaths from malaria having almost halved since 2000 [1]. The scale of this success reinforces the notion that the complete global eradication of malaria may be an attainable goal. With this in mind, the pharmacological properties of antimalarial drugs have recently been regarded from a different perspective [2] – in addition to curing symptomatic infections, the broader public health implications with regards to their role in reducing ongoing malaria transmission at the population level must also be considered.

The success of malaria elimination strategies will fundamentally rest on interrupting the cycle of parasite transmission between the Anopheles mosquito vector and humans. Strategies can target not only transmission from mosquito to human but also from human to mosquito, prompting renewed interest both in so-called “transmission-blocking” human malaria vaccines and the role of antimalarial drugs for preventing transmission to mosquitoes.

**Fig. 1:**
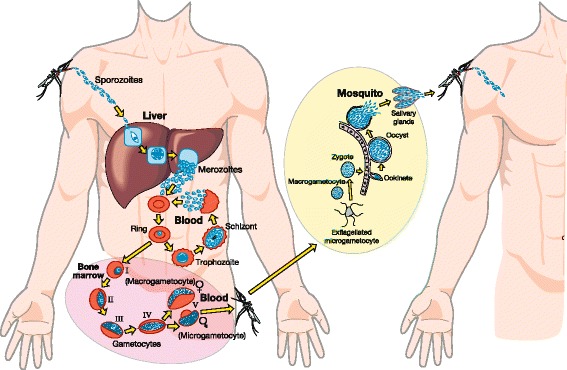
The life cycle of *Plasmodium falciparum*, demonstrating how onward transmission occurs from one human host to the next. Drugs with “transmission-blocking” properties can act on any of the sexual stages of the parasite (gametocytes from stage I to V) occurring within the human host (*pink-shaded area*). However, because a mosquito’s blood meal will contain concentrations of any drug present at the time of biting, drugs administered to humans can also have transmission-blocking potential through their activity on stages present in the mosquito mid-gut (*yellow-shaded area*)

Gametocytes arise mainly as progeny of “asexual” blood-stage parasites (merozoites). The maturation of *Plasmodium falciparum* gametocytes within humans has been divided into five morphologically and transcriptionally distinct stages (Fig. 1) [3]. Only the most mature stage 5 gametocytes are infectious to mosquitoes and, following ingestion, travel to the mosquito mid-gut to develop into gametes. If both male and female gametes are present, sexual reproduction ensues, leading ultimately to sporozoites able to infect humans. Therefore, gametocytes are imperative to human-to-mosquito transmission for all malaria species. *P. falciparum* gametocytes have a highly distinctive morphology, meaning that they can easily be detected, distinguished from other life-stages, and quantified using conventional microscopic techniques. The finding of gametocytes on a standard Giemsa-stained blood film (together with metrics derived from their concentration over time) has therefore been used as an easily generated indicator of an individual’s potential for onward transmission of malaria [4, 5].

## Some antimalarial drugs clear gametocytes from the blood more rapidly than others

Antimalarial drugs differ in their relative activity on the various life-cycle stages of the malaria parasite, a property often termed “stage specificity”, which can manifest through the more effective gametocytocidal activity of certain drugs over others [6]. Although new in vitro techniques can assess drug gametocytocidal activity [6], in vivo data is needed to establish drug activity within the human host.

Artemisinin combination therapies (ACTs) are now the mainstay in the treatment of most malaria cases worldwide [7]. Five ACTs, each utilizing a different “partner drug” are currently approved for use. It could be argued that differences in gametocytocidal activity between these ACTs could have important public health implications.

In a research article published in *BMC Medicine* [8], the WWARN investigators identify clear differences between different ACTs with regards to gametocyte clearance. They show that those with 4-aminoquinoline partner drugs (dihydroartemisinin-piperaquine and artesunate-amodiaquine) clear gametocytes significantly slower than those with aryl-amino alcohol and related structures (artesunate-mefloquine and artemether-lumefantrine). These data infer that, whilst 4-aminoquinoline-based ACTs may be perfectly effective in curing an individual patient’s clinical malaria infection, they could be inferior from a public health perspective given their reduced effectiveness at preventing onward transmission. It could be argued that artesunate-mefloquine and artemether-lumefantrine have advantages for malaria control, especially if elimination is the ultimate aim. Others have also argued that primaquine, a drug with potent gametocytocidal activity, should be routinely deployed as adjunctive therapy to further reduce transmission following ACT treatment [9].

## How much does gametocyte clearance actually really matter?

There are important reasons why the differences in gametocyte clearance identified by the WWARN investigators should be interpreted with caution. Firstly, microscopic evidence of gametocytaemia may not necessarily be as accurate an indicator of human-to-mosquito transmissibility as might be expected. Indeed, microscopy cannot distinguish viable, living parasites from those that are dead or have been affected by a drug in a way that compromises their infectivity [10, 11]. Further, routine microscopy usually does not distinguish between the most mature infective forms and the less mature non-infective forms. Therefore, considering that drugs may have differing gametocytocidal activity at the various stages of development, simply measuring the total numbers of gametocytes can be misleading [4]. Finally, microscopy is relatively insensitive and methods based on reverse-transcriptase quantitative PCR can unmask both a much larger potentially infective population and longer durations of potential infectivity following treatment [4]. In combination, these and other factors indicate that there is no simple linear relationship between gametocyte density and true infectivity. Indeed, some individuals with little or no gametocytemia are infectious and others with very high gametocytemia are non-infectious. Therefore, microscopy-based metrics of blood gametocyte clearance kinetics can only be considered “indirect” or “surrogate” markers of true infectivity to mosquitoes.

Ideal “gold standard” tests should rely on experiments known as direct (membrane) feeding assays. These require mosquitoes to be allowed to feed on human subjects (or at least on fresh human blood through an artificial membrane), after which mosquito mid-guts are dissected to determine whether oocysts have been formed. Yet, being labor-intensive and requiring significant insectary and laboratory facilities in a malaria endemic setting, it is not surprising that data from definitive studies of this type are lacking. Nevertheless, when such studies have been performed, they have generated some surprising and counterintuitive findings. For example, a previously widely-held belief was that, based on in vitro and in vivo observations of very high and prolonged gametocyte prevalence following treatment with the antimalarial sulfadoxine-pyrimethamine, this drug was a particularly bad culprit for facilitating onward transmission. However, direct feeding experiments showed these gametocytes are, in fact, poorly infective [10, 11]. Another recent study evaluating adjunctive gametocytocidal treatment (primaquine added to artemether-lumefantrine) showed that only one of 49 subjects in the control group (who received only artemether-lumefantrine without primaquine) was able to infect mosquitoes following treatment despite over 30 % having microscopically detectable post-treatment gametocytemia [12]. This data challenges recent calls for routine deployment of adjunctive primaquine gametocytocidal treatment following ACT case management [9] and demonstrates how important direct infectivity data are for assessing the effect of different treatments on malaria transmission.

## Uncertainties in transmissibility within sub-populations

Furthermore, there is a singular, crucial, unresolved epidemiological question that must be urgently addressed. In endemic settings, at any one time, the total transmissible biomass can be thought of as residing in three separate sub-populations: (1) those who have become unwell with malaria, but have yet to be treated; (2) those who have been treated and may remain infectious for a variable period (depending on drug and other factors); and (3) the population of chronic asymptomatic carriers (who are unlikely to receive antimalarial treatment at all). The relative contribution of each group to overall transmission will depend on three factors that may vary according to the epidemiological setting: (1) the size of each group relative to one another; (2) the duration for which individuals in each group remain infectious; and (3) how infectious individuals in each group are relative to those in the other groups. In other words, what is the probability that a single mosquito feed will result in human-mosquito transmission and how does this vary between groups? In the absence of good data from direct feeding experiments, this last factor remains essentially unclear. For instance, because asymptomatic carriers have generally lower concentrations of circulating gametocytes (often at sub-microscopic levels) it is uncertain just how infectious they are. However, because they usually represent the largest of the three groups and since they may have (potentially) much longer durations of infectivity (months or even years, compared with days or weeks in the other two groups) asymptomatic carriers could possibly represent a substantial contributor to human-mosquito transmission in many epidemiological settings. One mathematical modeling study suggests this could be to such a degree that post-treatment gametocytaemia (in the second group) becomes irrelevant [13]. However, such modeling exercises rely on assumptions regarding the infectivity of asymptomatic carriers that are open to debate. These uncertainties can only be resolved with robust infectivity data from direct feeding studies that include asymptomatic carriers living in endemic areas.

## Conclusions

The WWARN investigators have again demonstrated the impressive power of pooling data from a large number of studies and how this can unearth significant between-treatment differences in pharmacodynamic endpoints that would not ordinarily be detected. However, we must accept that its findings are subject to limitations inherent both in their use of an imperfect, indirect, surrogate biomarker for transmission and our currently limited understanding of the relative contribution of symptomatic and asymptomatic infections under different transmission scenarios. We must therefore be careful to distinguish the statistical significance of its findings from their actual clinical or public health implications. To this effect, we must first address fundamental gaps in our knowledge of the biology and epidemiology of malaria transmission through field studies using direct feeding assays, including in asymptomatic carriers. These studies are difficult and resource intensive to perform, but not impossible. The significant investment required is more than justified by the returns they are likely to generate for guiding policy and future research in malaria.

## Abbreviations

ACT, artemisinin-based combination therapy; WWARN, Worldwide Antimalarial Resistance Network
